# Sign Tracking, but Not Goal Tracking, is Resistant to Outcome Devaluation

**DOI:** 10.3389/fnins.2015.00468

**Published:** 2015-12-16

**Authors:** Sara E. Morrison, Michael A. Bamkole, Saleem M. Nicola

**Affiliations:** ^1^Department of Psychiatry and Behavioral Science, Albert Einstein College of MedicineBronx, NY, USA; ^2^Dominick P. Purpura Department of Neuroscience, Albert Einstein College of MedicineBronx, NY, USA

**Keywords:** sign tracking, goal tracking, devaluation, Pavlovian conditioning, reward, rats

## Abstract

During Pavlovian conditioning, a conditioned stimulus (CS) may act as a predictor of a reward to be delivered in another location. Individuals vary widely in their propensity to engage with the CS (sign tracking) or with the site of eventual reward (goal tracking). It is often assumed that sign tracking involves the association of the CS with the motivational value of the reward, resulting in the CS acquiring incentive value independent of the outcome. However, experimental evidence for this assumption is lacking. In order to test the hypothesis that sign tracking behavior does not rely on a neural representation of the outcome, we employed a reward devaluation procedure. We trained rats on a classic Pavlovian paradigm in which a lever CS was paired with a sucrose reward, then devalued the reward by pairing sucrose with illness in the absence of the CS. We found that sign tracking behavior was enhanced, rather than diminished, following reward devaluation; thus, sign tracking is clearly independent of a representation of the outcome. In contrast, goal tracking behavior was decreased by reward devaluation. Furthermore, when we divided rats into those with high propensity to engage with the lever (sign trackers) and low propensity to engage with the lever (goal trackers), we found that nearly all of the effects of devaluation could be attributed to the goal trackers. These results show that sign tracking and goal tracking behavior may be the output of different associative structures in the brain, providing insight into the mechanisms by which reward-associated stimuli—such as drug cues—come to exert control over behavior in some individuals.

## Introduction

Sign tracking describes the propensity of individuals to engage with a conditioned stimulus (CS) that has been paired with a rewarding unconditioned stimulus (US), even when the location of the eventual reward is not colocalized with the CS (Hearst and Jenkins, 1974; Flagel et al., 2009). It is often thought to be a manifestation of Pavlovian conditioned approach (PCA), and thus driven by the incentive salience that has been attributed to the CS (Berridge, 2004; Anselme et al., 2013; Robinson et al., 2014). Many studies have shown broad individual variation in sign tracking behavior (Robinson and Flagel, 2009), with some subjects engaging almost exclusively with the CS (e.g., a light or a lever), while other subjects engage with the site of reward (e.g., a food trough or receptacle). This latter behavior is referred to as goal tracking (Boakes, 1977).

Sign tracking paradigms have recently gained in popularity as a model for examining how certain environmental stimuli—e.g., drug cues—gain powerful influence over behavior in some individuals but not others (Flagel et al., 2009). An individual's tendency to sign track is predictive of several measures of drug-seeking (Tomie et al., 2008; Saunders and Robinson, 2011; Meyer et al., 2012b; Robinson et al., 2014), as well as of impulsive approach to reward-associated cues, including drug-related stimuli (Flagel et al., 2010; Saunders and Robinson, 2013). Moreover, only in sign trackers, as opposed to goal trackers, will a reward-associated CS become an effective secondary reinforcer, such that rats will work to receive the CS even without the original rewarding outcome (Robinson and Flagel, 2009). These observations are thought to indicate that sign trackers have a higher propensity to transfer incentive salience to a CS.

Sign tracking and goal tracking both require associative learning between a cue and an outcome; and for both sign trackers and goal trackers, the CS becomes an effective driver of behavior. However, it has been proposed that sign tracking and goal tracking behaviors, while both driven by associative learning, are propelled by different representational structures within the brain (Clark et al., 2012; Huys et al., 2014; Lesaint et al., 2014). One structure, represented by goal tracking, is the association between the CS and an explicit representation of the outcome, which results in behavior specific to the properties of the US (e.g., its spatial location). An alternative structure, represented by sign tracking, is the association between a CS and the motivational properties of the outcome, resulting in the CS acquiring incentive value of its own.

Indirectly supporting this two-structure framework is the finding that rats bred to preferentially sign track show a transference of the dopamine signal from the US, in early stages of learning, to the CS, as the animal learns to approach the CS. Selectively bred goal-trackers show no such transfer (Flagel et al., 2011b). Similarly, sign trackers (but not goal trackers) develop elevated levels of dopamine in the nucleus accumbens (NAc; Tomie et al., 2000), and sign tracking (but not goal tracking) is profoundly sensitive to dopamine receptor antagonism within the NAc (Saunders and Robinson, 2012). Together, these results suggest that sign tracking is the result of activity in the mesolimbic dopamine system, perhaps because dopamine serves to assign incentive salience to reward-associated objects (Berridge and Robinson, 1998; Berridge, 2004); in contrast, goal-tracking appears to be independent of mesolimbic dopamine.

Despite these observations, there is little direct evidence that, as predicted by theory (Clark et al., 2012; Huys et al., 2014; Lesaint et al., 2014), the CSs that are the objects of sign tracking acquire incentive value in a manner that is independent of the outcome. It is conceivable that some or all of the behaviors assumed to be “Pavlovian” responses, including PCA and sign tracking, are actually linked to the specific features of the US, as has been shown for certain forms of conditioned approach (Holloway and Domjan, 1993; Hilliard and Domjan, 1995). In order to test these alternate hypotheses, we designed an experiment to examine whether devaluation of the outcome—a liquid sucrose reward—influences sign tracking behaviors, goal tracking behaviors, or both. Here, we provide direct evidence that sign tracking behavior, as it is commonly studied, is indeed resistant to changes in the value of the outcome, whereas goal tracking behavior—at least in rats that preferentially goal-track—is profoundly sensitive to the value of the outcome.

## Materials and methods

All procedures were performed in accordance with the National Institutes of Health *Guide for the Care and Use of Laboratory Animals* and were approved by the Institutional Animal Care and Use Committee of the Albert Einstein College of Medicine.

### Subjects

Subjects were 48 naïve adult male Long-Evans rats obtained from Charles River Laboratory. The study was run in three replications involving 16 rats each. Rats weighed 275–300 g upon arrival. They were singly housed and placed on a 12 h light/dark cycle; all experiments were conducted during the light phase. After arrival, rats were allowed to acclimate to the housing colony for at least 7 d. They were then habituated to human contact and handling over 2 or 3 days before the start of training. Behavioral training took place over 9–17 days (with a schedule of 5 days on, 2 days off). This was followed by two consecutive days for the devaluation protocol (during which rats did not perform the task) and 1 day for the extinction test and sucrose consumption trial.

Subjects were provided with water *ad libitum* throughout, and food (standard chow) was provided *ad libitum* until the start of training. After acclimation and habituation, rats were placed on a restricted diet of 15 g of chow per day (BioServ F-137 dustless pellets), which continued throughout behavioral training and experiments. Rats were weighed daily to ensure they maintained a minimum of 90% of pre-restriction body weight.

### Apparatus

All training and experiments took place in standard operant chambers measuring approximately 30 × 25 cm (Med Associates; St. Albans, VT, USA) housed inside sound-attenuating cabinets equipped with ventilating fans. The chambers were illuminated with two 28 V white house lights and, at all times during the experiment, white noise (≈65 dB) was played from a dedicated speaker, masking outside noise. Another speaker was used for playing auditory cues during the task. One wall of the chamber contained a reward receptacle flanked by two retractable levers; only one lever was used for each rat, counterbalanced across subjects. Blue cue lights were located above each lever. The behavioral task was controlled by MED-PC software (Med Associates) and behavioral events (lever deflections and receptacle entries/exits) were collected at a resolution of 1 ms.

### Training

Rats were trained using a Pavlovian conditioned approach procedure similar to those used by others (e.g., Tunstall and Kearns, 2015). Each training session began with the illumination of the house lights. Rats were initially trained over 2 days to retrieve rewards (drops of 10% liquid sucrose) from the receptacle in the absence of predictive stimuli. Each receptacle training session consisted of 50 rewards delivered on a variable interval schedule averaging 90 s.

Rats then completed either 15 training sessions (16 rats) or 7 training sessions (32 rats). After completion of training for the first cohort of 16 rats, we chose to reduce the number of sessions for subsequent cohorts because behavior tended to become relatively stable after approximately 5 sessions. Results from the first cohort and subsequent cohorts were substantially similar (data not shown), so we combined data from all groups. The first cohort contributed roughly evenly to the operationally defined sign tracker and goal tracker populations (9 sign trackers and 7 goal trackers; see Results).

Training sessions consisted of trials separated by an intertrial interval chosen from a truncated exponential distribution averaging 60 s. Each trial was initiated by presentation of the CS: extension of either the left or right lever (counterbalanced among subjects) for 8 s accompanied by flashing of the corresponding cue light (the CS-on period). After 8 s, the lever retracted, the cue light extinguished, and the reward was delivered into the receptacle, accompanied by a 1 s auditory cue (intermittent 6 kHz tone, approximately 85 dB). No action was required in order for reward to be delivered. During each training session, 25 trials were presented.

### Reward devaluation and testing

**Figure 1:**
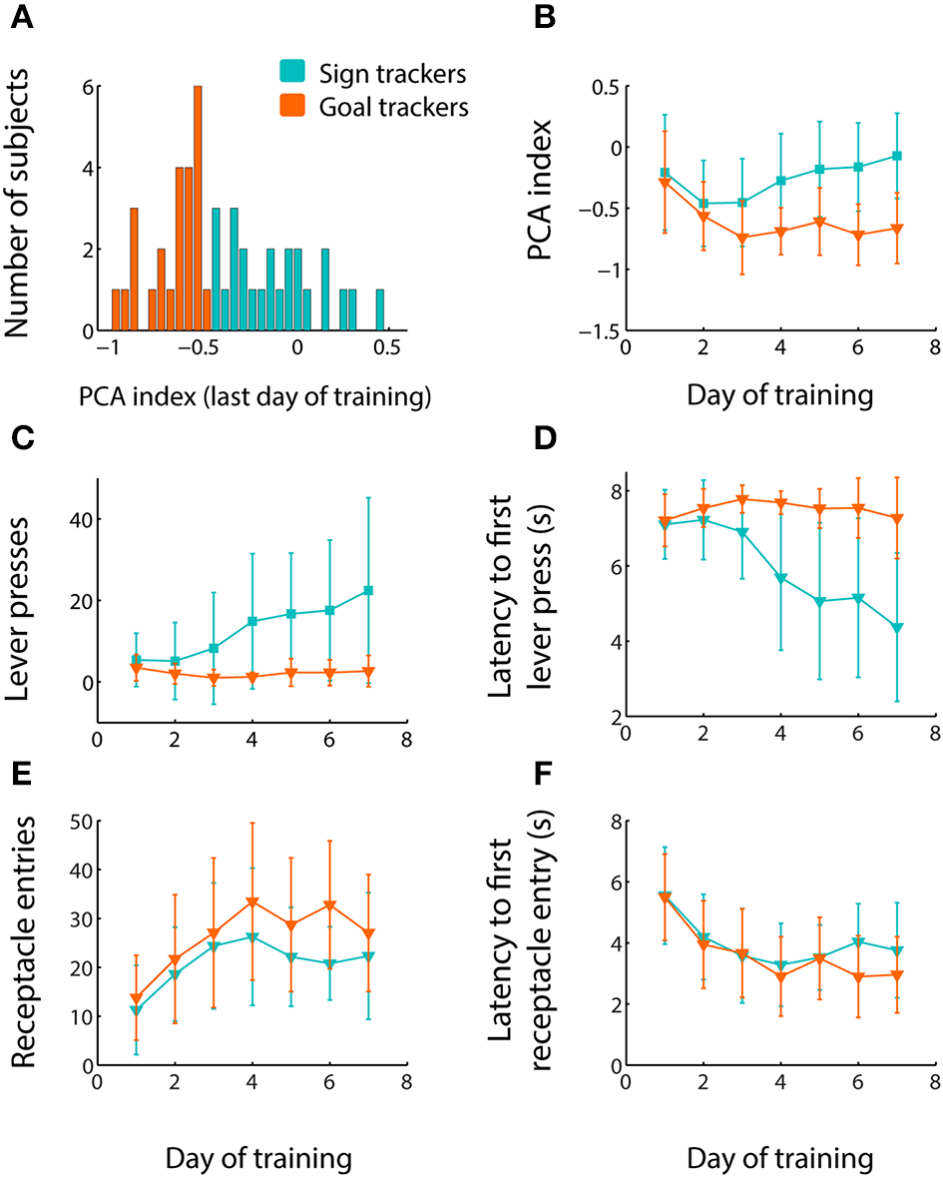
**Individual subjects exhibit wide variation in sign-tracking behavior. (A)** PCA index (see Materials and Methods for details of calculation) for all 48 subjects on the last day of training. “Goal trackers” (orange) and “sign trackers” (blue), for the purposes of this study, are rats with PCA index below or above the median, respectively. **(B–F)** Mean PCA index **(B)**, mean total number of lever presses during CS-on periods **(C)**, mean latency to first lever press during the CS-on period **(D)**, mean total number of receptacle entries during CS-on periods **(E)**, and mean latency to first receptacle entry during the CS-on period **(F)**, all ±SEM, over the first 7 days of training for goal trackers (orange) and sign trackers (blue).

Devaluation of the sucrose US was accomplished by using taste aversion conditioning. Approximately 24 h after the last day of training, rats were divided into two behavior-matched groups based on a composite “PCA index” calculated for each subject during the last training session (see below and Figure 1A). The two groups were then subjected to reward devaluation (“paired” group) or sham devaluation (“unpaired” group). Paired rats were given access to 20 mL of 10% sucrose in the home cage for 20 min. Immediately after consumption, all rats in both groups were injected with lithium chloride (LiCl; 0.3 or 0.6 M; 0.5 mL/kg ip). We found that behavioral effects were not markedly different with these two doses of LiCl (data not shown), so we combined data across doses for all analyses. Approximately 24 h later, unpaired rats were given access to sucrose. Immediately after consumption, all rats in both groups were given an equivalent volume injection of 0.9% saline. In this way, both groups received similar home cage sucrose exposure and the same number and type of injections.

On the day following saline injections, rats were given a test session in extinction. The test session was identical to the training sessions, except it consisted of only 20 trials and no rewards were delivered. Finally, rats were given a sucrose consumption test 1–3 h after completion of the test session, consisting of 20 min of access to 20 mL of 10% sucrose in their home cages.

### Data analysis

All analyses were carried out using custom written programs in Matlab (Natick, MA, USA). Analyses focused on the first 10 trials of the extinction test and, by way of comparison, the first 10 trials of each of the four final days of training. We chose to focus on the first half of the test session in order to highlight the effects of reward devaluation in contrast to the effects of extinction on behavioral responding.

We quantified the degree to which rats engaged in sign-tracking vs. goal tracking behavior during CS presentation by using a Pavlovian Conditioned Approach (PCA) index (Saunders and Robinson, 2011; Meyer et al., 2012a), which comprises the average of three ratios: (1) the probability index, which compares the probability of lever deflection vs. receptacle entry during the 8 s CS-on period, calculated as (P_lever_ – P_receptacle_), (2) the bias index, which compares the average total number of lever deflections and receptacle entries per CS-on period, calculated as (#lever − #receptacle)/(#lever + #receptacle), and (3) the latency index, which compares the average latency from CS onset to lever deflection vs. latency from CS onset to receptacle entry on each trial, calculated as (receptacle latency − lever latency)/(CS length). For trials in which a behavior was not performed, the latency for that behavior was defined as the total CS length (i.e., 8 s). All of these indices range from −1.0 to +1.0 and are more positive for animals that preferentially sign tack (i.e., interact with the lever) and are more negative for animals that preferentially goal track (i.e., interact with the receptacle). Therefore, the PCA index also ranges from −1.0 to 1.0 and is more positive for sign trackers and more negative for goal trackers.

In addition to the PCA index and its components, we also developed a “First Response” index (FR index) of sign tracking, which reports the proportion of all trials (including no-response trials) in which the subject's initial response to the CS is a lever deflection.

All statistical comparisons were performed using a Wilcoxon signed rank test (within-group comparisons) or a Wilcoxon rank sum test (across-group comparisons). Where appropriate, *p*-values are reported after correction for multiple comparisons by the Holm-Sidak method; an adjusted *p* < 0.05 was considered significant, and *p* < 0.1 (adjusted and/or unadjusted) was considered a trend toward significance. All regressions are linear and computed using the least-squares method.

## Results

We trained 48 rats on a standard Pavlovian procedure in which the CS (lever extension accompanied by a flashing cue light) terminated with delivery of a liquid sucrose reward in a receptacle that was spatially distinct from the CS.

### Acquisition of sign tracking and goal tracking behavior

In this task, which is similar to other paradigms commonly used to study sign tracking and related behaviors (e.g., Meyer et al., 2012a; Tunstall and Kearns, 2015), sign tracking is represented by interactions with the lever during the CS (lever deflections) and goal-tracking is represented by interactions with the reward receptacle during the CS (receptacle entries). Importantly, neither of these behaviors was required for obtaining the reward after CS termination. We quantified individual rats' propensity to sign track or goal track by calculating a PCA index (see Materials and Methods) ranging from −1.0 (all goal-tracking behavior, no sign-tracking behavior) to +1.0 (all sign-tracking behavior, no goal-tracking behavior). Figure 1A shows the PCA index of all individuals on the last day of training. There was broad variation in the relative levels of sign tracking and goal tracking in individual rats, although all rats exhibited goal tracking behavior to some degree, and no rats showed exclusive or near-exclusive sign tracking. This behavior profile is reflected in the fact that the distribution of PCA indices was skewed to the negative.

The broad and negative distribution of PCA indices did not readily reveal distinct populations of “sign trackers” and “goal trackers,” as in some previous studies in which the distribution was more bimodal (e.g., Saunders and Robinson, 2011). Therefore, for some analyses, we divided rats into two groups based on whether each individual's PCA index on the last day of training was above or below the median of −0.466: a relatively low sign-tracking group (orange in Figure 1) and relatively high sign-tracking group (blue in Figure 1). Operationally, we will refer to these groups as “goal trackers” and “sign trackers” respectively, although the sign tracker group clearly includes some individuals that might have been classified as goal trackers (or intermediates) in other studies. Figures 1B,C illustrate the acquisition of sign tracking and goal tracking behaviors over the course of the first 7 days of training. The average PCA index, which quantifies overall sign tracking relative to goal tracking, gradually increased in the sign tracker group, while remaining low and stable in the goal tracker group (Figure 1B). This was mainly driven by an increase in number of lever presses (Figure 1C), and a decrease in latency to first lever press (Figure 1D), during the CS-on period among the sign tracker group over the course of training. In contrast, both groups increased their number of receptacle entries over the course of training—with the goal tracking group achieving a slightly higher number at asymptote (Figure 1E)—and decreased their latency to first receptacle entry (Figure 1F).

All rats learned to rapidly and consistently enter the receptacle to collect the reward after the termination of the cue: over the last 4 days of training, rats entered the receptacle within 10 s after cue offset on 97.1 ± 0.5% of trials with a latency averaging 0.59 ± 0.07 s. These measures did not differ between operationally defined sign trackers and goal trackers (reward collection probability: *Z* = 1.80, *p* > 0.05; reward collection latency: *Z* = 0.30, *p* > 0.1).

### Reward devaluation increases sign tracking relative to goal tracking

After completion of Pavlovian training, rats were divided into two behavior-matched groups (based on the PCA index from the last training session). The “paired” group underwent a reward devaluation procedure via induction of a taste aversion: exposure to the US (10% sucrose in the home cage) was paired with illness via injection of LiCl. The “unpaired” group underwent a sham devaluation procedure in which sucrose exposure was paired with vehicle injection. Both groups received equivalent sucrose exposure in the home cage, and both groups received both LiCl and vehicle injections (see Materials and Methods); but only in the “paired” group was sucrose temporally linked to illness.

**Figure 2:**
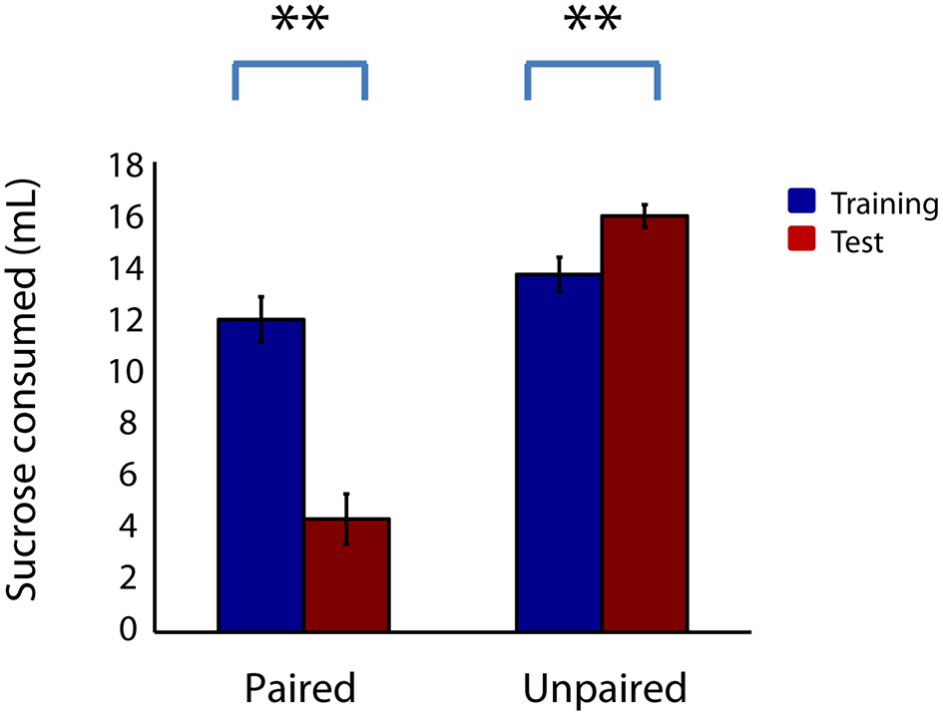
**Free sucrose consumption is markedly reduced following reward devaluation**. Sucrose solution (10%) consumed over 20 min of exposure in the home cage for the paired group (left) and unpaired group (right) before (blue) and after (red) the devaluation or sham devaluation procedure. Error bars, ±SEM. Double asterisks, corrected *p* < 0.001.

The devaluation procedure successfully induced an aversion to sucrose, as shown in Figure 2. During a consumption test approximately 48 h after devaluation, the paired group showed drastically decreased sucrose consumption (*Z* = −3.89, *p* < 0.001). Many rats in the paired group consumed no sucrose at all during 20 min of exposure. Rats in the unpaired group, in contrast, consumed slightly more sucrose during the consumption test than prior to sham devaluation (*Z* = −3.29, *p* < 0.05).

Following the devaluation/sham devaluation procedure (and before the consumption test), rats were given a test session of the Pavlovian paradigm in which no rewards were given (i.e., in extinction). Importantly, rats did not experience the taste of sucrose, or the relationship between the CS and sucrose, between the devaluation procedure and the test session. Therefore, any changes in CS-evoked behavior we observed must be explained by a representational cognitive process incorporating the updated value of the US.

**Figure 3:**
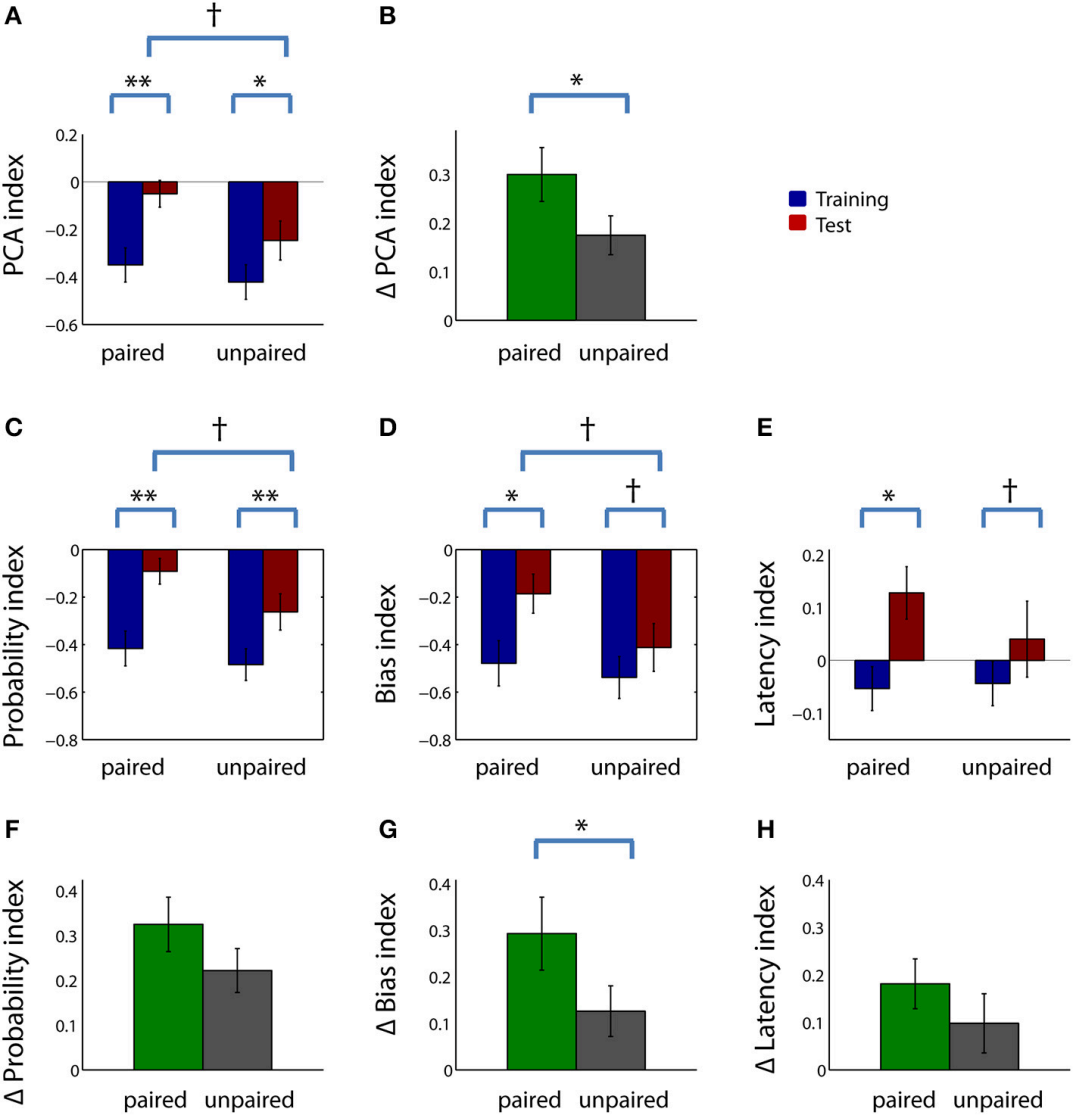
**Sign-tracking behavior is increased, not decreased, relative to goal-tracking behavior in rats that have undergone reward devaluation**. **(A)** Mean PCA index during the last 4 days of training (left) and in the test session (right) for the paired group (blue) and unpaired group (red). **(B)** Mean change in PCA index from training to test session for the paired group (green) and unpaired group (gray). **(C–E)** Mean probability index **(C)**, bias index **(D)**, and latency index **(E)** for the paired group (left) and unpaired group (right) during the last 4 days of training (blue) and during the test session (red). **(F–H)** Mean change in probability index **(F)**, bias index **(G)**, and latency index **(H)** from training to test session for the paired group (green) and unpaired group (gray). See Materials and Methods for details of index calculation. All panels: error bars, ±SEM; double asterisks, corrected *p* < 0.001; single asterisk, corrected *p* < 0.05; dagger, corrected or uncorrected *p* < 0.1. All unlabeled comparisons are non-significant (uncorrected *p* > 0.1).

We first compared the behavior of the devalued and control groups as a whole (without dividing them into sign- and goal-trackers). Within the extinction test session, rats in the paired group tended to have a higher PCA index, on average, than rats in the unpaired group (Figure 3A; *Z* = 1.82, uncorrected *p* < 0.1), indicating that the paired group had a higher ratio of sign tracking behavior to goal tracking behavior. The evidence for this was stronger when we took into account variations in baseline responding: rats in the paired group showed a large increase in PCA index between the last four training sessions and the test session (Figure 3A; *Z* = −3.66, *p* < 0.001), while rats in the unpaired group showed a smaller (but still significant) increase (*Z* = −3.40, *p* < 0.05). Of note, we would expect most rats to have some level of increase in PCA index in the test session because it is done in extinction, causing a reduction in receptacle entries as rats learn that the CS no longer predicts reward. However, the increase in PCA index in the paired group was significantly larger than the increase in the unpaired group (Figure 3B; *Z* = 1.99, *p* < 0.05). There was no difference in the magnitude of the increase in PCA index among cohorts with different numbers of training sessions in either the paired group (*Z* = 0.64, *p* > 0.1) or the unpaired group (*Z* = 0.46, *p* > 0.1).

Next, we looked more closely at behavioral changes in the paired and unpaired groups by examining the individual components of the composite PCA index (Figures 3C–H). For each of the three measures, rats in the paired group showed significant increases in sign-tracking behavior relative to goal-tracking behavior, whereas rats in the unpaired group showed smaller changes. Compared to training, in the test session, rats in the paired group had a higher probability of lever deflection relative to receptacle entry during the CS (Figure 3C; *Z* = −3.65, *p* < 0.001); a larger bias toward lever deflection over receptacle entry during the CS (Figure 3D; *Z* = −3.09, *p* < 0.05); and a shorter relative latency to lever deflection (Figure 3F; *Z* = −2.71, *p* < 0.05). Rats in the unpaired group showed a significantly higher probability index (Figure 3C; *Z* = −3.59, *p* < 0.001)—likely reflecting the change from rewarded to unrewarded conditions in the test session—but only trends toward a higher bias index (Figure 3D; *Z* = −2.28, corrected *p* < 0.1) and a higher latency index (Figure 3E; *Z* = −2.02, uncorrected *p* < 0.1). The increase in the bias index was significantly greater in the paired group than the unpaired group (Figure 3G; *Z* = 1.05, *p* < 0.05), although the differences in the other indices did not attain significance. Notably, though, the paired group attained a significantly positive latency index after devaluation (*p* < 0.05, signed rank test)—indicating that rats in this group generally interacted with the lever before interacting with the receptacle—whereas the unpaired group's latency index was not significantly different from zero (*p* > 0.1).

**Figure 4:**
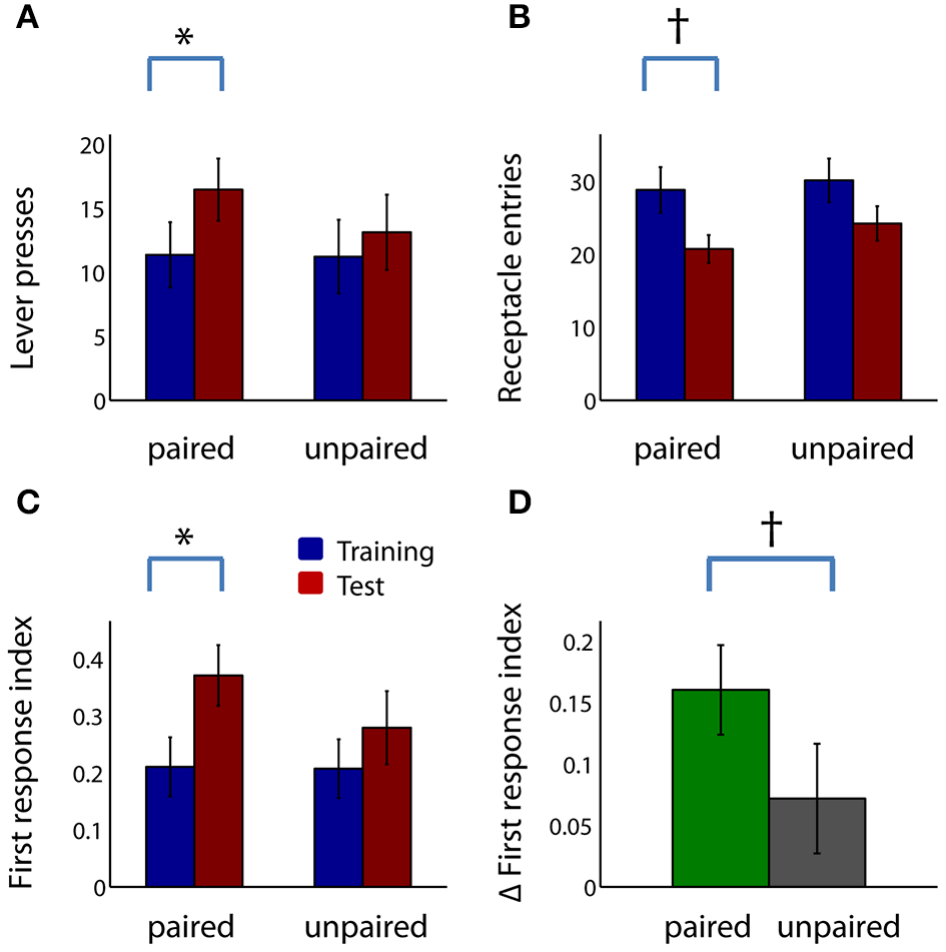
**Reward devaluation increases the proportion of trials in which subjects respond to the CS with a lever press**. **(A,B)** Mean lever deflection count **(A)** and receptacle entry count **(B)** during the CS-on periods for the paired group (left) and unpaired group (right) in the last 4 days of training (blue) and in the test session (red). **(C)** Mean first response index for the paired group (left) and unpaired group (right) during the last 4 days of training (blue) and the test session (red). See Materials and Methods for details of index calculation. **(D)** Mean change in first response index from training to test session for the paired group (green) and the unpaired group (gray). All panels: error bars, ±SEM; asterisk, corrected *p* < 0.05; dagger, corrected or uncorrected *p* < 0.1. All unlabeled comparisons are non-significant (uncorrected *p* > 0.1).

Thus, by several measures, the ratio of sign tracking to goal tracking behavior was greater in the paired group than in the unpaired group, and/or showed greater increases from training to test session. Inspection of the raw behavioral counts indicated that the greater increase in these indices in the paired group was driven by both a significant increase in lever deflections (Figure 4A; *Z* = −2.51, *p* < 0.05) and a trend toward a decrease in receptacle entries (Figure 4B; *Z* = −1.67, corrected *p* < 0.1). Neither lever deflections nor receptacle entries were significantly different after sham devaluation in the unpaired group.

The decrease in relative latency to interact with the lever vs. the receptacle (Figure 3E) suggested that, after devaluation, the lever CS exerted more attractive power, relative to the reward receptacle, and therefore rats might more often approach the CS as their initial response to CS onset. To directly investigate this possibility, we examined the proportion of trials in which lever deflection was the first (or only) behavioral event during CS presentation (first response index; Figures 4C,D). The first response index was significantly increased after reward devaluation for the paired group (Figure 4C; *Z* = −3.36, *p* < 0.05) but not the unpaired group (*Z* = −1.01, *p* > 0.1). (The first response index did not surpass 0.5, contrary to what the latency index might suggest, because the latency index does not take into account trials in which neither response occurred). Although unpaired rats also showed a slight increase in the first response index, the increase tended to be larger for rats in the paired group (Figure 4D; *Z* = 1.80, uncorrected *p* < 0.1). Thus, as indicated by a variety of measures, devaluation of the rewarding outcome augments sign tracking behavior and decreases goal tracking behavior.

### Reward devaluation primarily affects subjects with a goal-tracking bias

Because we observed a wide spectrum of individual differences in sign-tracking and goal-tracking propensity (Figure 1), we wondered whether subjects that showed a greater degree of interaction with the CS (“sign trackers”) were more or less likely to change their behavior after reward devaluation than subjects that preferentially interacted with the site of reward (“goal trackers”). Differences in the sensitivity of sign- and goal-trackers to the effects of devaluation could explain why we sometimes observed only marginal effects of devaluation in the population as a whole (e.g., Figures 3C,E,F,H). Therefore, as shown in Figure 1A, we divided subjects into those relatively more likely to sign track (sign trackers) and those less likely to sign track (goal trackers). Using this operational definition, there were an equal number of sign tracker and goal tracker rats (*n* = 12 of each) in the paired and unpaired groups.

**Figure 5:**
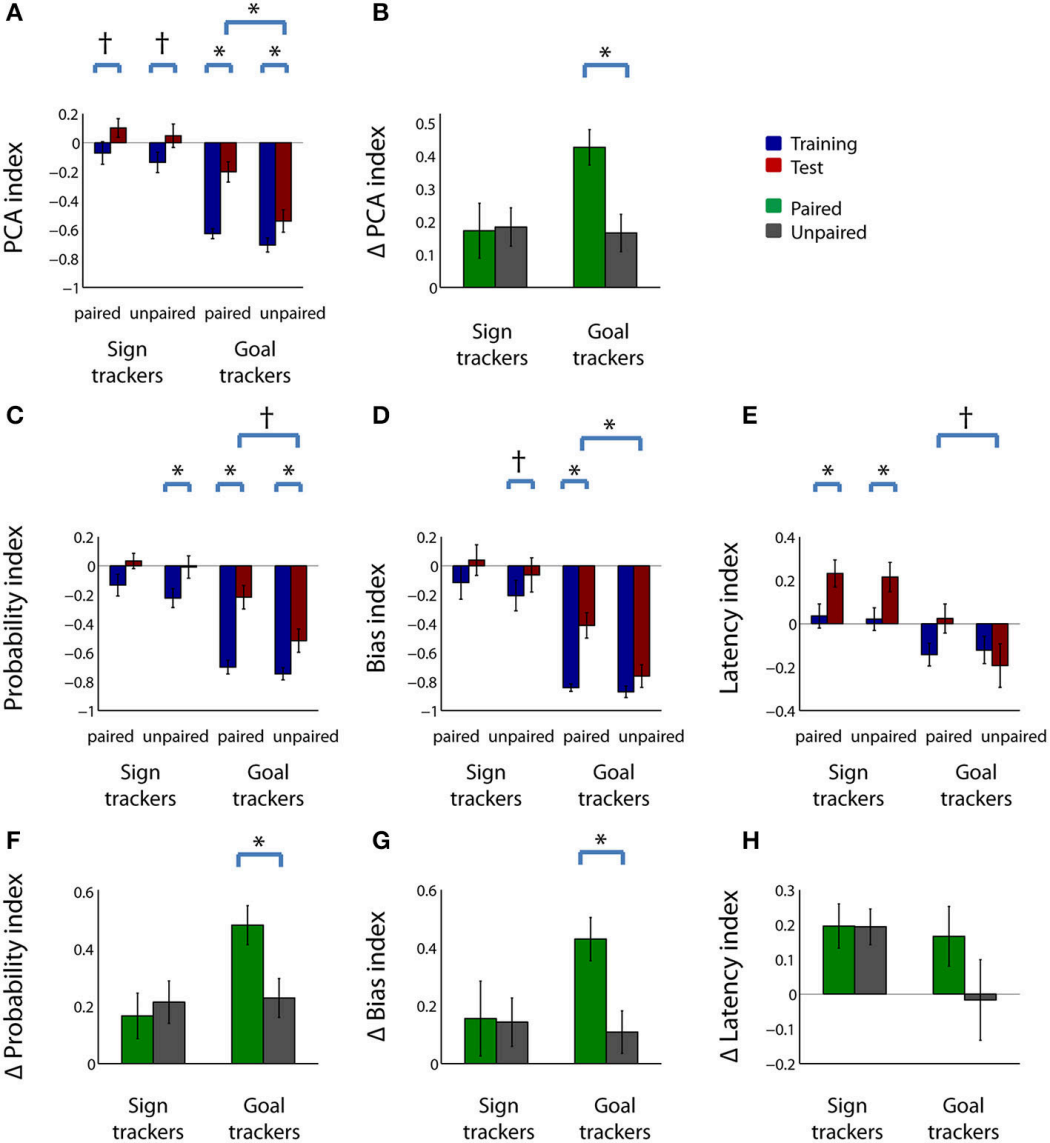
**Goal trackers, and not sign trackers, are responsible for virtually all changes in CS response behavior following reward devaluation**. **(A)** Mean PCA index for operationally defined sign trackers (left-hand side of panel) and goal trackers (right-hand side of panel) in the paired group (blue) and unpaired group (red) during the last 4 days of training (“pre”) and the test session (“post”). **(B)** Mean change in PCA index from pre- to post-devaluation for sign trackers (left) and goal trackers (right) in the paired group (blue) and unpaired group (red). Both panels: error bars, ±SEM; single asterisk, *p* < 0.05; n.s., non-significant (*p* > 0.1). **(C–E)** Mean probability index **(C)**, bias index **(D)**, and latency index **(E)** for operationally defined sign trackers (left-hand side of panels) and goal trackers (right-hand side of panels) during the last 4 days of training (blue) and the test session (red) in the paired group and unpaired group. **(F–H)** Mean change in probability index **(F)**, bias index **(G)**, and latency index **(H)** from training to test session among sign trackers (left) and goal trackers (right) for the paired group (green) and unpaired group (gray). All panels: error bars, ±SEM; asterisk, corrected *p* < 0.05; dagger, corrected or uncorrected *p* < 0.1. All unlabeled comparisons are non-significant (uncorrected *p* > 0.1).

In the test session after the devaluation procedure, we observed a significantly higher PCA index (i.e., a shift toward proportionally greater sign tracking) in the paired group than the unpaired group in the goal tracker subjects only (Figure 5A; *Z* = 2.63, *p* < 0.05). Moreover, while both sign tracker and goal tracker rats exhibited the expected increase in PCA index in the extinction session, as compared to the last four training sessions, only among goal tracker rats was this increase significantly (and dramatically) greater in the paired than the unpaired group (Figure 5B; *Z* = 2.86, *p* < 0.05).

Examination of the individual components of the PCA index revealed that, after reward devaluation, paired goal tracker rats, compared to unpaired goal tracker rats, showed a trend toward greater probability of lever deflection relative to receptacle entry during the CS (Figure 5C; *Z* = 2.10, corrected *p* < 0.1); a significantly greater bias toward lever deflection over receptacle entry during the CS (Figure 5D; *Z* = 2.63, *p* < 0.05); and a trend toward lower latency to interact with the lever, relative to the receptacle (Figure 5E; *Z* = −1.67, uncorrected *p* < 0.1). None of these measures were significantly different between paired and unpaired sign tracker rats (uncorrected *p* > 0.1); nor were any of the measures significantly different between paired and unpaired groups prior to devaluation (uncorrected *p* > 0.1). Although sign trackers also exhibited increases in some of these measures during the test session, only among goal trackers was the increase in any of the indices significantly greater in the paired than the unpaired group (Figures 5F–H; probability index, *Z* = 2.28, *p* < 0.05; bias index, *Z* = 2.80, *p* < 0.05).

**Figure 6:**
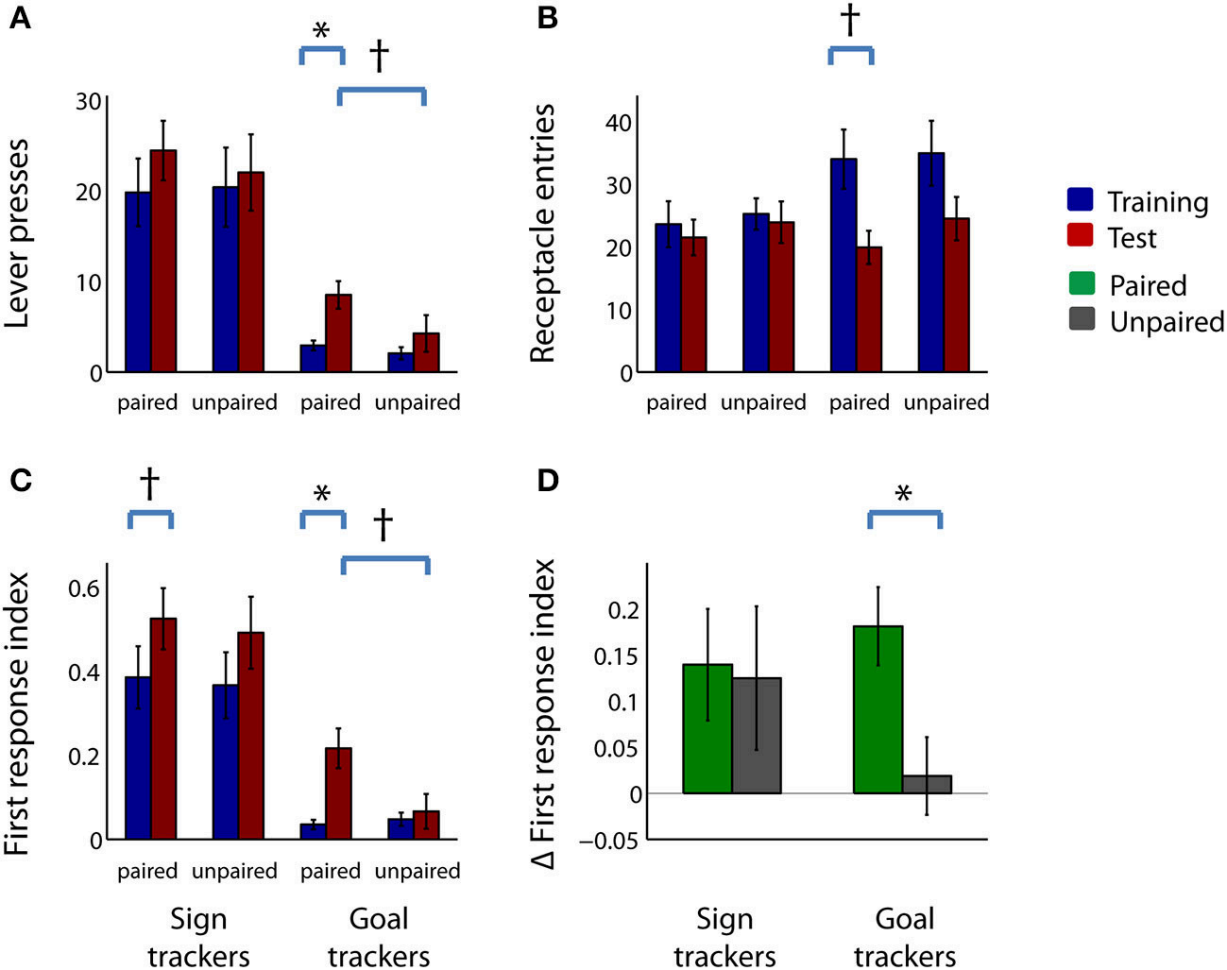
**In goal trackers, but not sign trackers, reward devaluation increases the proportion of trials in which subjects respond to the CS with a lever press**. **(A,B)** Mean lever deflection count **(A)** and receptacle entry count **(B)** among operationally defined sign trackers (left-hand side of panel) and goal trackers (right-hand side of panel) in the last 4 days of training (blue) and in the test session (red) for the paired group and unpaired group. **(C)** Mean first response index for sign trackers (left) and goal trackers (right) during the last 4 days of training (blue) and the test session (red) for the paired group and unpaired group. **(D)** Mean change in first response index from training to test session for sign trackers (left) and goal trackers (right) in the paired group (green) and unpaired group (gray). All panels: error bars, ±SEM; asterisk, corrected *p* < 0.05; dagger, corrected or uncorrected *p* < 0.1. All unlabeled comparisons are non-significant (uncorrected *p* > 0.1).

Next, we examined the raw behavior counts for sign trackers and goal trackers. The counts show that, just as in the larger population, in paired goal tracker rats, the post-devaluation change in the PCA index and its components was driven by both an increase in lever presses (Figure 6A; *p* < 0.05) and a trend toward a decrease in receptacle entries (Figure 6B; corrected *p* < 0.1). In contrast, neither of these behavior counts was significantly different before and after devaluation in sign tracker rats (uncorrected *p* > 0.1), even those that had undergone the devaluation procedure. Interestingly, unlike goal tracker rats, sign tracker rats (paired as well as unpaired) showed little decrease in receptacle entries even when rewards were no longer available (median change in receptacle entries not different from zero, *p* > 0.1, signed rank test).

Among goal tracker rats—but not sign tracker rats—devaluation markedly increased the proportion of trials in which a lever deflection was the initial response to CS onset (Figures 6C,D). During the test session, the first response index trended higher in paired goal tracker rats than unpaired goal tracker rats (Figure 6C; *Z* = 2.29, corrected *p* < 0.1), but not for paired sign tracker rats compared with unpaired sign tracker rats (uncorrected *p* > 0.1). Moreover, when we compared the last training sessions and the test session, only among goal tracker rats was the increase in first response index significantly greater in the paired than the unpaired group (Figure 6D; *Z* = 2.73, *p* < 0.05). Overall, these results imply that virtually all of the behavioral changes seen following reward devaluation in the paired group as a whole (Figures 3, 4) can be attributed specifically to a shift toward proportionally greater sign tracking in the goal tracker subpopulation, whereas the sign tracker subpopulation showed no such shift.

**Figure 7:**
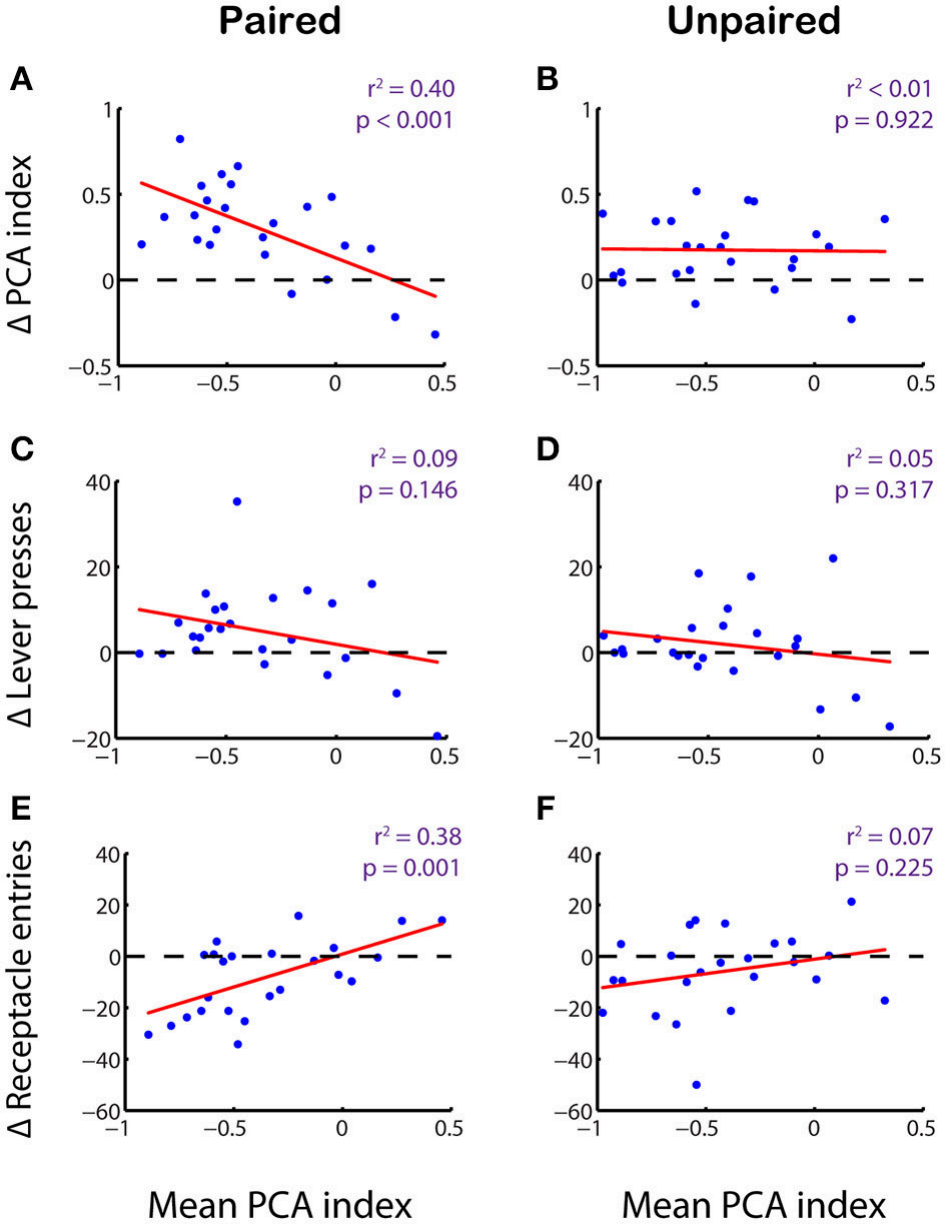
**The effectiveness of reward devaluation varies with individual subjects' propensity to goal-track**. PCA index of individual subjects during training (average over last 4 sessions) plotted against change in PCA index **(A,B)**, total lever presses during CS-on periods **(C,D)**, or total receptacle entries during CS-on periods **(E,F)** between training and test session for the paired **(A,C,E)** and unpaired **(B,D,F)** groups. Regression lines in red.

Finally, we examined whether the effects of reward devaluation are related to individual differences in propensity to sign track or goal track on a subject-by-subject basis. Indeed, we found that the PCA index of individual rats—averaged over the last 4 days of training—was strongly correlated with the change in PCA index seen in the test session (Figure 7A; *r*^2^ = 0.40, *p* < 0.001). This correlation was specific to the effects of devaluation: in the unpaired group, no such correlation was observed (Figure 7B; *r*^2^ < 0.001, *p* = 0.922), even though most individuals showed a positive change in PCA index from training to test session. Just as in the goal tracker population as a whole (Figure 6B), the stronger effect of devaluation on individuals with a higher propensity to goal track was mainly driven by a decrease in receptacle entries following devaluation (Figure 7E; *r*^2^ = 0.38, *p* = 0.001), sometimes accompanied by a moderate increase in lever pressing (Figure 7C; *r*^2^ = 0.09, *p* = 0.146). The unpaired group, in contrast, showed no correlation between individual propensity to sign track or goal track and changes in either lever pressing (Figure 7D; *r*^2^ = 0.05, *p* = 0.317) or receptacle entries (Figure 7F; *r*^2^ = 0.07, *p* = 0.225).

When we examined devaluation effects on the components of the PCA index, we observed robust correlations in the paired group between initial PCA index and the change in the probability index (*r*^2^ = 0.45, *p* < 0.001) and the bias index (*r*^2^ = 0.23, *p* = 0.017), although not the latency index (*r*^2^ = 0.01, *p* = 0.690); no such correlations were present for the unpaired group (probability index: *r*^2^ = 0.004, *p* = 0.755; bias index: *r*^2^ < 0.001, *p* = 0.892; latency index: *r*^2^ = 0.12, *p* = 0.118). Thus, sign-tracking behavior (lever pressing) is insensitive to reward devaluation, whereas goal-tracking behavior (as measured by number of receptacle entries) is robustly affected by reward devaluation in a manner that depends on subjects' individual propensity to engage in goal-tracking rather than sign-tracking behavior.

## Discussion

In the present study, we used reward devaluation to interrogate the content of the CS-reward association underlying sign-tracking behavior. We found that reward devaluation does not decrease sign-tracking behavior in a classic Pavlovian paradigm. On the contrary, devaluation of the rewarding outcome (liquid sucrose) markedly increases measures of sign tracking relative to goal tracking—a change that can be attributed mainly to a decrease in goal-tracking behavior (head entries into the reward receptacle), but also to an increase in sign-tracking behavior (interaction with the lever CS) that was possibly compensatory. These findings are complementary to two recent studies reporting that sign-tracking behavior is far less vulnerable to extinction than goal-tracking (Ahrens et al., 2015; Beckmann and Chow, 2015). Together with the current work, these studies provide strong experimental support for the hypothesis that sign tracking reflects a transfer of incentive salience from the reward to the CS via a “model-free” process (Clark et al., 2012; Huys et al., 2014; Robinson et al., 2014)—i.e., an associative structure that does not include the specific characteristics of the US.

These data imply that sign-tracking behavior, as commonly studied in the lab, is independent of neural representations of the US and its specific properties, including its current value and its relationship to the CS. It should be noted, however, that the specific properties of the US might still come into play during the initial formation of the conditioned response (CR): it has long been observed that the specific form of the CR can depend strongly on, e.g., the modality of the US (Davey and Cleland, 1982). In addition, these data do not imply that the CS does not or cannot evoke an explicit representation of the US in the brain—only that such a representation does not control sign-tracking behavior.

As in many previous studies, we were interested in possible differences between individuals that preferentially interacted with the site of reward (“goal trackers”) and individuals that tended to interact with the lever CS (“sign trackers”). Interestingly, we found that reward devaluation strongly and selectively affected the behavioral output of operationally defined goal trackers, but not sign trackers. In fact, nearly all of the behavioral changes observed in the wider population following devaluation could be attributed to the goal tracker subpopulation, with the greatest contribution from those individuals with the highest levels of goal tracking relative to sign tracking. Again, this was primarily the result of a decrease in goal-tracking behavior, which was sometimes abetted by a complementary increase in sign tracking. We conclude that goal-tracking behavior (at least among goal tracker individuals) is profoundly sensitive to the properties of the US, including its updated value and whether the relationship between the CS and the US is currently valid (as shown in this study as well as Ahrens et al., 2015; Beckmann and Chow, 2015). The observation that goal tracking behavior is strongly affected by reward devaluation—even before the subject has experienced the devalued reward in conjunction with the CS—lends further support to the idea that goal tracking emerges from a “model-based” learning system involving an explicit representation of the US (Gallagher et al., 1999; Pickens et al., 2003; Clark et al., 2012).

Under the current experimental paradigm, we observed a wide spectrum of sign-tracking behavior relative to goal-tracking behavior (Figure 1A). However, if we applied the criteria of some previous studies, we found relatively few individuals that would have been categorized as sign trackers (PCA index > 0; as used by, e.g., Saunders and Robinson, 2011; Meyer et al., 2012a; Ahrens et al., 2015). This seems to be due to the persistence of receptacle entries even in individuals with high levels of lever-oriented behavior; i.e., most of the “sign trackers” continued to exhibit substantial amounts of “goal tracking” (discussed further below). Moreover, unlike some authors (e.g., Saunders and Robinson, 2011), we did not find a bimodal distribution of the PCA index; instead, as in other studies (e.g., Tunstall and Kearns, 2015), we observed a broad spectrum of behavioral phenotypes. In some previous studies (e.g., Ahrens et al., 2015), authors have dealt with such a spectrum by eliminating from analysis subjects with an “intermediate” PCA index. For the purposes of the present study, we chose to preserve as much information as possible by simply splitting subjects along the median PCA index and comparing the resulting “high sign tracking” and “low sign tracking” (i.e., goal tracking) subpopulations.

There are several differences from previous studies that might contribute to the ubiquity of goal tracking behavior—and the absence of “pure” sign trackers—among our subject population. First, we chose a liquid reward (10% sucrose solution) instead of the grain or sucrose pellets used in most previous studies of sign-tracking and goal-tracking behaviors. The use of a liquid reward is common among neurobiological studies of reward-based learning in rodents and primates (e.g., Morrison and Salzman, 2009; Morrison and Nicola, 2014) because it facilitates electrophysiological recording (by reducing chewing artifacts); therefore, it will be invaluable in future studies of the neural circuits that underlie sign tracking and goal tracking. However, the slightly different form of the US—e.g., the fact that it is not easily separable from the receptacle, physically or conceptually—might have altered the facility with which is becomes associated with the receptacle relative to the lever. Moreover, many studies have shown that the form of the US influences the specific conditioned response (CR) developed to the associated CS—e.g., licking the lever when the US is a liquid vs. biting the lever when the US is a solid (Davey and Cleland, 1982)—and it is possible that the latter CR might result in more lever deflections than the former. In the present study, we have no way of discerning the specific form of the CR.

Secondly, we employed an ancillary stimulus—a 1 s auditory tone—that coincided with the delivery of reward. We found that this stimulus facilitated training by (presumably) helping rats understand the temporal connection between CS offset and reward delivery; but it is possible that the tone increased the salience of the reward delivery event, helping to enhance the receptacle-reward association at the expense of the CS-reward association. Alternatively, or in addition, the tone might have taken on some of the incentive salience that would otherwise have been credited to the lever, thereby reducing lever-focused behavior.

A third difference is the strain of rats used: our subjects were Long-Evans rats, whereas most prior studies—with a few key exceptions (e.g., Chang and Holland, 2013; Tunstall and Kearns, 2015)—have used Sprague-Dawley rats. Several authors have noted differences in sign-tracking propensity among different rat strains (e.g., Kearns et al., 2006). Even among Sprague-Dawley rats, in addition to variation in sign tracking among individuals, there is a wide degree of variation across different populations: e.g., from different vendors, or even among different colonies from the same vendor (Fitzpatrick et al., 2013). In fact, consistent with the current findings, Fitzpatrick et al. found that rats obtained from Charles River (the source of the subjects used here) included more classically defined goal trackers than sign trackers.

Whatever the underlying cause, the persistence of “goal-tracking” behavior among sign tracker rats—even those with the highest level of lever deflections—led us to an interesting observation: among sign tracker individuals (but not goal tracker individuals), interactions with the reward receptacle appeared to be unaffected by reward devaluation. This leads us to the hypothesis that, under some conditions and in some subjects—i.e., individuals prone to sign tracking—the receptacle itself can acquire incentive salience, perhaps in compound with other nearby cues. By this reasoning, some or all of the receptacle entries performed by sign tracker rats during the CS are more similar to sign tracking than to goal tracking in their underlying representational structure. In other words, the fact that the subject interacts with the “sign” or the “goal” does not, by itself, directly indicate that a model-free representational system controls the former behavior and a model-based system controls the latter. Instead, additional evidence—such as that which can be obtained by reward devaluation—is required to establish the degree to which a neural representation of the outcome influences each behavior.

Just as the current data imply that some behavior commonly thought of as “goal tracking” may actually stem from a model-free associative structure, there may also be situations in which “sign tracking” behavior can come under the control of a model-based structure. For example, one classic study (Cleland and Davey, 1982) found that under a more complex set of circumstances—two levers, two rewarding outcomes, and multiple reversals of contingencies—lever pressing was indeed diminished upon reward devaluation. Consistent with this finding is a preliminary report that sign tracking is susceptible to reward devaluation when two comparable but distinguishable rewards are associated with two different levers during the same session (Derman and Delamater, 2014). In both of these contexts, the relative complexity of the task—e.g., the cognitive load demanded by tracking multiple contingencies—might have resulted in the predomination of a model-based representation in the brain.

Meanwhile, a growing body of evidence implicates the NAc, and especially its mesolimbic dopamine input, in model-free reinforcement learning and promotion of sign tracking-like behaviors. Markers for dopamine (Tomie et al., 2000) and for general neural activity (Flagel et al., 2011a) in mesolimbic areas are increased during sign tracking, and NAc dopamine surges during CS presentation in sign tracker rats (Flagel et al., 2011b). Furthermore, sign-tracking (but not goal-tracking) behavior can be blocked by dopamine receptor antagonist injection in the NAc (Di Ciano et al., 2001; Flagel et al., 2011b; Saunders and Robinson, 2012) or by dopamine depletion of the NAc (Parkinson et al., 2002). We have recently identified a potential neural mechanism whereby NAc dopamine could promote such behaviors. Many NAc neurons are excited by cues that elicit approach (Nicola et al., 2004; Yun et al., 2004; Ambroggi et al., 2008, 2011). These excitations precede the onset of approach movement and predict its latency (McGinty et al., 2013); in addition, they are greatest when the subject is closest to the approach target rather than when the outcome value is greatest at that target (Morrison and Nicola, 2014). These signals are therefore precisely what we would expect of a neural representation of the incentive salience of the approach target (resulting from a model-free process), rather than a representation of the associated outcome (resulting from a model-based process).

Furthermore, cue-evoked excitations in the NAc are strongly dependent on dopamine and are essential for the subject to engage in cued approach behavior (du Hoffmann and Nicola, 2014). Therefore, we hypothesize that dopaminergic facilitation of cue-evoked excitations in the NAc underlies the model-free neural associative structure that drives sign tracking and other outcome-independent forms of PCA. This proposal rests on the assumption that the cued approach behaviors observed in the electrophysiological studies described above were under the control of a model-free rather than model-based representational system—an assumption that must be tested by determining how outcome devaluation affects task performance. Indeed, preliminary evidence suggests that devaluation of the sucrose reward does not impair extinction performance of the standard “discriminative stimulus” task used for these studies (Meffre et al., 2015).

Establishing that sign-tracking behavior is resistant to reward devaluation and extinction—particularly in the Pavlovian paradigms commonly used to study addiction and impulsivity—is an essential step toward understanding how the objects of PCA, such as drug cues, come to powerfully control behavior in ways that are often maladaptive (Everitt and Robbins, 2005; Flagel et al., 2010). Moreover, the current work provides essential experimental grounding for theoretical accounts of sign tracking and goal tracking, particularly the recent hypothesis that Pavlovian conditioned responses can be controlled by both model-based and model-free representational structures (Clark et al., 2012; Lesaint et al., 2014). Ultimately, a sound understanding of the neural mechanisms underlying these dual computational processes will be invaluable in identifying and treating those individuals most vulnerable to impulse-control disorders such as drug addiction.

## Author contributions

SM and SN designed the experiment and wrote the paper. MB and SM carried out the experiment. SM analyzed the data.

### Conflict of interest statement

The authors declare that the research was conducted in the absence of any commercial or financial relationships that could be construed as a potential conflict of interest.
